# The presence of tumour-associated lymphocytes confers a good prognosis in pancreatic ductal adenocarcinoma: an immunohistochemical study of tissue microarrays

**DOI:** 10.1186/1471-2407-13-436

**Published:** 2013-09-24

**Authors:** Nilanjana Tewari, Abed M Zaitoun, Arvind Arora, Srinivasan Madhusudan, Mohammad Ilyas, Dileep N Lobo

**Affiliations:** 1 Division of Gastrointestinal Surgery, Nottingham Digestive Diseases Centre National Institute for Health Research Biomedical Research Unit, Nottingham University Hospitals, Queen’s Medical Centre, Nottingham NG7 2UH, UK; 2 Department of Cellular Pathology, Nottingham University Hospitals, Queen’s Medical Campus, Nottingham NG7 2UH, UK; 3 Academic Oncology, University of Nottingham, School of Molecular Medical Sciences, Nottingham NG5 1PB, UK; 4 Nottingham University Hospitals, City Hospital Campus, Nottingham NG5 1PB, UK; 5 Division of Academic Pathology, University of Nottingham, Queen’s Medical Centre, Nottingham NG7 2UH, UK

**Keywords:** CD3, CD8, Pancreatic ductal adenocarcinoma, Tumour-associated lymphocytes, Prognosis, Immunohistochemistry

## Abstract

**Background:**

Tumour-associated lymphocytes (TALs) have been linked with good prognosis in several solid tumours. This study aimed to evaluate the prognostic significance of CD3, CD8 and CD20 positive lymphocytes in pancreatic ductal adenocarcinoma.

**Methods:**

After histological re-evaluation of the tumours of 81 patients who underwent surgical resection for exclusively pancreatic ductal adenocarcinoma, tissue micro-arrays (TMA) were constructed and immunohistochemistry was performed for CD3, CD8 and CD20. The number of lymphocytes within specific tumour compartments (i.e. stromal and intratumoural) was quantified. X-tile software (Yale School of Medicine, CT, USA) was used to stratify patients into 'high’ and 'low’ for each of the lymphocytes stained and their association with survival. Receiver operating curves (ROC) were constructed to evaluate the association between the TALs, alone and in combination, with clinicopathological features.

**Results:**

CD3 and CD8 positive lymphocytes were associated with grade of tumour differentiation. The presence of intratumoural CD3 positive cells was associated with improved survival (p = 0.028), and intratumoural and stromal CD3 in combination also correlated with improved survival (p = 0.043). When CD20 positive lymphocyte levels were high, survival improved (p = 0.029) and similar results were seen for CD20 in combination with intratumoural CD3 (p = 0.001) and stromal CD8 (p = 0.013).

**Conclusions:**

This study has shown a correlation between the presence of TALs and survival in pancreatic ductal adenocarcinoma.

## Background

Pancreatic cancer is one of the most aggressive of gastrointestinal malignancies, with a 5- year survival rate of 4-5% [1,2]. Surgical resection offers the only potential for cure, but no more than 20% of pancreatic carcinomas are resectable [2]. Chemotherapy may be offered for unresectable disease, but in patients with locally advanced or metastatic disease, progression-free survival time after chemotherapy is usually less than 3 months [3]. Despite advances in surgical technique and reduction in postoperative mortality, the death rate for pancreatic cancer has remained relatively stable [4].

It has long been recognised that the tumour microenvironment has an important role in the biological behaviour of cancer [5]. The host response to presence of tumour cells can be represented by the presence of tumour infiltrating immune cells. This reaction has been evaluated in a number of solid tumours and several tumour associated lymphocytes (TALs) have been studied [5-8].

The CD3 antigen is a 20 kD glycoprotein, present on the surface of all human T lymphocytes [9]. CD8 is a two chain glycoprotein which is expressed on the surface of circulating T lymphocytes. CD3 is a generic T-cell receptor and will identify all T-cells while CD8 is a marker for cytotoxic T-cells and suppressor T cells [10]. In the first paper to analyse the profile of tumour infiltrating lymphocytes [11], CD3, CD8 and some other markers were evaluated and it was found that both CD3 and CD8 were associated with prognosis. Staining for CD3 and CD8 has been used in non-small cell lung cancer [12], colorectal cancer [11] and cutaneous lymphoma [13] to demonstrate the prognostic significance of TALs. Many other studies have used a similar strategy and in a recent meta-analysis of the role of TALs [14], it was concluded that CD3 and CD8 were associated with good prognosis.

CD20 is a 33-37 kDa transmembrane phosphoprotein which is expressed on B lymphocyte precursors and mature B lymphocytes [15,16]. CD20 positive B cell infiltration has been associated with improved patient survival in primary breast cancer [16], non-small cell lung cancer [17] and epithelial ovarian cancer [18]. They have not previously been studied in cancers of the pancreas. This study evaluated the prognostic significance of CD3, CD8 and CD20 positive lymphocytes in pancreatic ductal adenocarcinoma.

## Methods

### Clinical samples

Patients who underwent surgery with curative intent for pancreatic ductal adenocarcinoma between 8/3/96 and 21/6/11 were included in this study. All operations were performed in the Department of Hepatopancreaticobiliary Surgery, Nottingham University Hospitals NHS Trust. In this centre, 30-50 patients are operated on per year for periampullary cancer. In this study, we included only pancreatic ductal adenocarcinomas. Therefore, tumours of the ampulla, neuroendocrine tumours, cholangiocarcinomas and any other histological tumour types were excluded. Types of operations performed were Whipple’s procedure, total pancreatectomy, and distal pancreatectomy for tumours in the body, head, neck and tail of pancreas. Median follow up time was 42 months (6 to 163 months). Ethical approval was obtained from the National Regional Ethics Service Committee East Midlands - Nottingham 1 for the use of anonymised archival specimens and the requirement for patient/relative consent was waived by the Ethics Committee. The study was conducted according to REMARK criteria [19].

### Tissue microarray and immunohistochemistry

Tissue microarrays (TMA) were prepared using triplicate 0.6 mm tissue cores of tumour, identified by a specialist pathologist (AMZ), placed into a single recipient paraffin block, using a semi-automated instrument and targeted cores. Sections of TMA 4 μm thick were mounted on poly-L-lysine coated slides.

Investigation of CD3, CD8 and CD20 was conducted using a tissue microarray. Immunohistochemical staining was performed using Bond Max automated staining machines (Leica Microsystems, Wetzlar, Germany). In our laboratory the antigen retrieval step is performed on our automated staining machines. The temperature at which the Leica Bond Max automated stainers retrieve is 100°C for the appropriate time (i.e.20 minutes) followed by 12 minutes at ambient temperature. CD3 (NCL-L-CD3-565, Leica Microsystems) was stained optimally at a dilution of 1/100 with antigen retrieval performed using Epitope Retrieval solution 2 (ER2) for 20 minutes. CD8 (NCL-L-CD8-295, Leica Microsystems) was stained optimally at a dilution of 1/50 with antigen retrieval performed using Epitope Retrieval solution 1 (ER1) for 30 minutes. CD20 (M0755, Dako) was stained optimally at a dilution of 1/400 with antigen retrieval performed using ER1 for 20 minutes. ER1 (AR9961, Leica Microsystems) is a ready-to-use citrate based pH 6.0 solution; ER2 (AR9640, Leica Microsystems) is a ready-to-use EDTA based pH 9.0 solution. Sections were cut, dried at room temperature for 20 minutes, then incubated at 60°C for 20 minutes prior to loading onto the Bond stainers (Leica Biosystems, Milton Keynes, UK). The Bond staining protocol comprises several steps. The first step is dewaxing using Leica Dewax solution (AR9222) for 30 seconds at 72°C, followed by the antigen retrieval step and application of the endogenous peroxidase block for 5 minutes at room temperature (RT). There follows primary antibody incubation for 15 minutes at RT, post primary incubation for 8 minutes at RT, polymer incubation for 8 minutes at RT, DAB for 10 minutes at RT, DAB enhancer (AR9432) for 5 minutes at RT and haematoxylin counterstain for 5 minutes at RT. Wash steps were performed using Leica Bond Wash solution (AR9590) between each step. Peroxidase blocks, post primary, polymer, DAB and Haematoxylin were all supplied in Leica Bond Refine Detection kit (DS9800). Sections were then removed from the Bond staining machine, dehydrated in IMS (Genta Medical, Rudgate, UK), cleared in Xylene (Genta Medical) and permanently mounted under glass coverslips using Pertex Histolab (Algol Diagnostics, Espoo, Finland). The sections are washed in Leica Bond wash buffer to rehydrate after the dewaxing step.

Evaluation of the number of CD3 and CD8 positive lymphocytes was performed as follows: the absolute number of each immunohistochemically detectable T-cell subgroup was counted manually by two independent researchers, one a pathologist. The localisation of each cell was taken into account – T lymphocytes lying among epithelial carcinoma cells were regarded as 'intratumoural’ while those within the tumour stroma were regarded as 'stromal’. The proportion of tumour and stroma was estimated visually by both researchers independently. From these figures, the densities of lymphocytes per mm^2^ were calculated using the TMA core area as reference [20]. The mean area of TMA cores was 0.355 mm^2^. All cases were scored without prior knowledge of pathological stage of tumour or patient outcome.

As CD20 staining was primarily seen in the stroma, only stromal B lymphocytes stained with CD20 were counted. The proportion of stroma was estimated by both researchers independently. These figures were used to calculate the density of CD20 positive B lymphocytes in the stroma using the TMA core area as reference. Therefore, CD20 results are not presented as 'intratumoural’ or 'stromal’ but simply as 'CD20’ which refers to CD20 positive TALs seen in stromal tissue.

### Statistical analysis

For inter-observer concordance, a subset of stained cores was scored by both observers and Kappa statistics were calculated to assess inter-observer variability. A subset of complete sections from patients in whom TMA sections had been evaluated was also scored by both observers. The mean number of TALs for each group resulting from the three tumour-TMA cores was attributed to the corresponding patient. The relationship between categorised protein expression and clinicopathological variables was assessed using Pearson Chi Square (*χ*^2^) test of association. Survival curves were plotted according to the Kaplan-Meier method and significance determined using the log-rank test. Multivariate survival analysis was performed by Cox Proportional Hazards regression model. All differences were deemed statistically significant at the level of *p* < 0.05. Statistical analysis was performed using SPSS 19.0 software (IBM Corporation, NY, USA).

X-tile software (Yale School of Medicine, CT, USA) was used to stratify patients into 'high’ and 'low’ for each of the lymphocytes stained. The use of X-tile has been described previously [21]. X-tile plots provide a single, global assessment of every possible way of dividing a population into low, medium, and high-level marker expression. X-tile data are presented in a triangular grid where each point represents a different cut-point. The intensity of the colour of each cut-off point represents the strength of the association. The X-tile software allows the user to move a cursor across the grid and provides a histogram of the resulting population subsets along with an associated Kaplan-Meier curve. This histogram can be used to determine the optimal cut-off point which shows up as the brightest pixel on the X-tile plot. The use of X-tile software allows results to be produced on a 'test’ cohort and tested on a 'validation’ cohort. Therefore, all survival results have been tested in a simulated independent cohort and found to be valid.

Stratification cut-points were determined using X-Tile software for survival analysis (Table 1) and receiver operating curves (ROC) for clinicopathological features and were determined prior to statistical analyses [21].

**Table 1:** Cut-offs for tumour associated lymphocytes and survival from X-tile

**Tumour associated lymphocytes**	**Cut-off score (cells/mm**^**2**^**)**
Intratumoural CD3	28.3
Stromal CD3	132.0
Intratumoural CD8	11.8
Stromal CD8	48.4
CD20	84.6

## Results

The study included 81 patients with pancreatic ductal adenocarcinoma. The median age of the patients was 65 years (range 25-82). The kappa statistic for inter-observer concordance was 0.78. On comparison of results of scoring whole tumour sections with TMA cores, there was no significant difference in mean score for intratumoural CD3 (p = 0.873), stromal CD3 (p = 0.895), intratumoural CD8 (p = 0.650), stromal CD8 (p = 0.436) or CD20 (p = 0.737). Clinicopathological data including tumour grade of differentiation are summarised in Table 2. Of note, there were no specimens in which lymph node status was pN2 so this is not mentioned in Table 2.

**Table 2:** Clinicopathological data

**Sex**	
Males	54 (66.7%)
Females	27 (33.3%)
**Location of tumour**	
Neck	1 (1.2%)
Body	4 (4.9%)
Tail	9 (11.1%)
Head	67 (82.7%)
**Resection Type**	
Total pancreatectomy	5 (6.2%)
Distal pancreatectomy/extended distal pancreatectomy and splenectomy	10 (12.3%)
Whipples’ procedure	66 (81.5%)
**pT category**	
pT2	16 (19.8%)
pT3	63 (77.8%)
pT4	2 (2.5%)
**pN category**	
pN0	24 (29.6%)
pN1	57 (70.4%)
**Tumour grade (G1 = well, G2 = moderately, G3 = poorly differentiated)**	
G1	8 (9.9%)
G2	45 (55.6%)
G3	28 (34.6%)
**CD3+ density [Mean (SEM), cells/mm**^**2**^**]**	
Intratumoural	42.4 (6.60)
Stromal	202.9 (25.9)
**CD8+ density [Mean (SEM), cells/mm**^**2**^**]**	
Intratumoural	15.2 (3.3)
Stromal	36.7 (3.7)
**Stromal CD20 density [Mean (SEM), cells/mm**^**2**^**]**	27.0 (5.2)

SEM = standard error of the mean.

### CD20, CD8 and CD3 expression in pancreatic tumours

**Figure 1:**
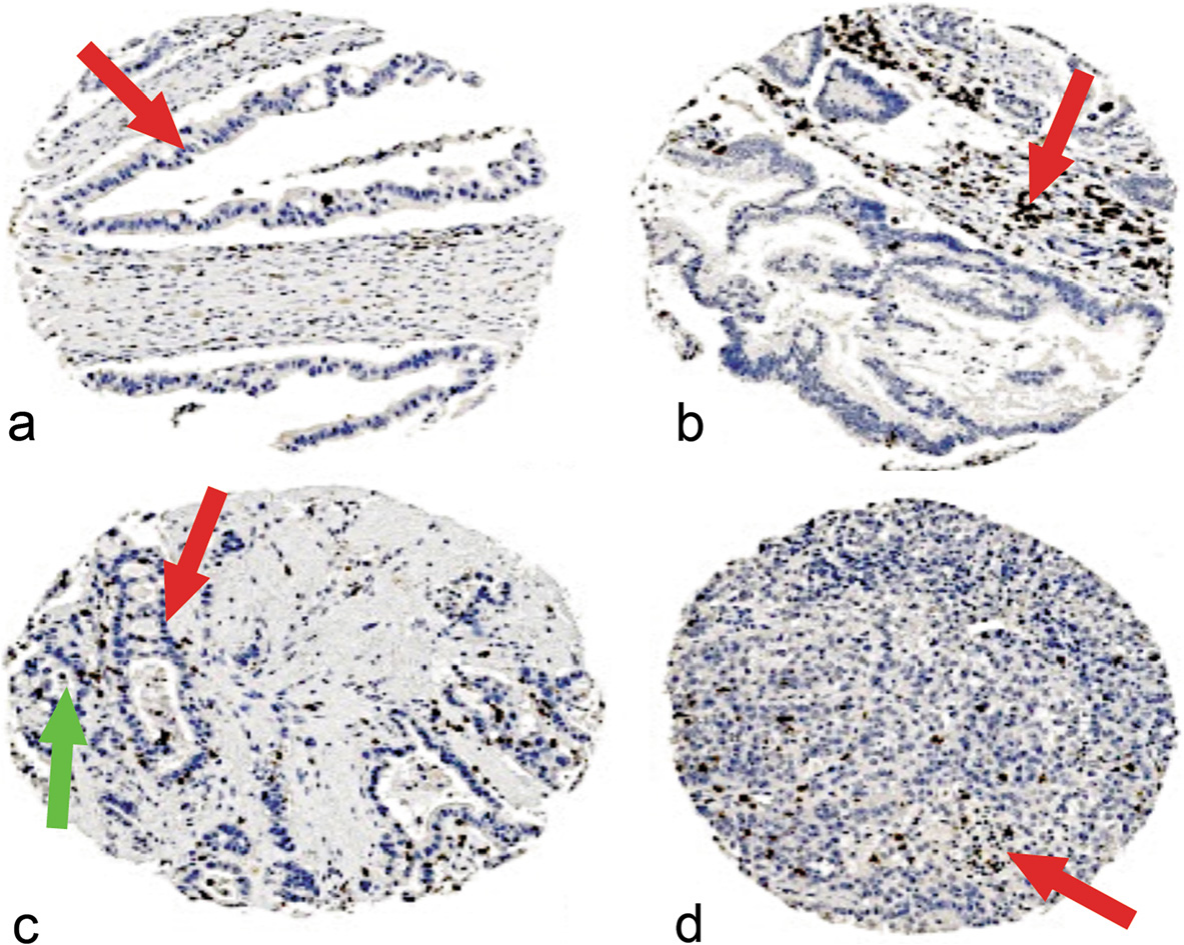
**Expression of CD3 in pancreatic ductal adenocarcinoma.** Representative photomicrographs of CD3 stained lymphocytes in pancreatic ductal adenocarcinoma. **a**: Small number of CD3 lymphocytes in both tumour epithelium (red arrow head) and stroma. **b**: Small number of CD3 lymphocytes in tumour epithelium and moderate number of CD3 lymphocytes in stroma (red arrow head) of well differentiated papillary adenocarcinoma of the pancreas (G1). **c**: Moderate number of CD3 lymphocytes in tumour epithelium (red arrow head) and occasional lymphocytes in stroma (green arrow head) of moderately differentiated adenocarcinoma (G2 ). **d**: Occasional CD3 lymphocytes in tumour epithelium and small number of CD3 lymphocytes in stroma (red arrow head) red of poorly differentiated papillary adenocarcinoma (G3). Photomicrographs are at 5× magnification.

**Figure 2:**
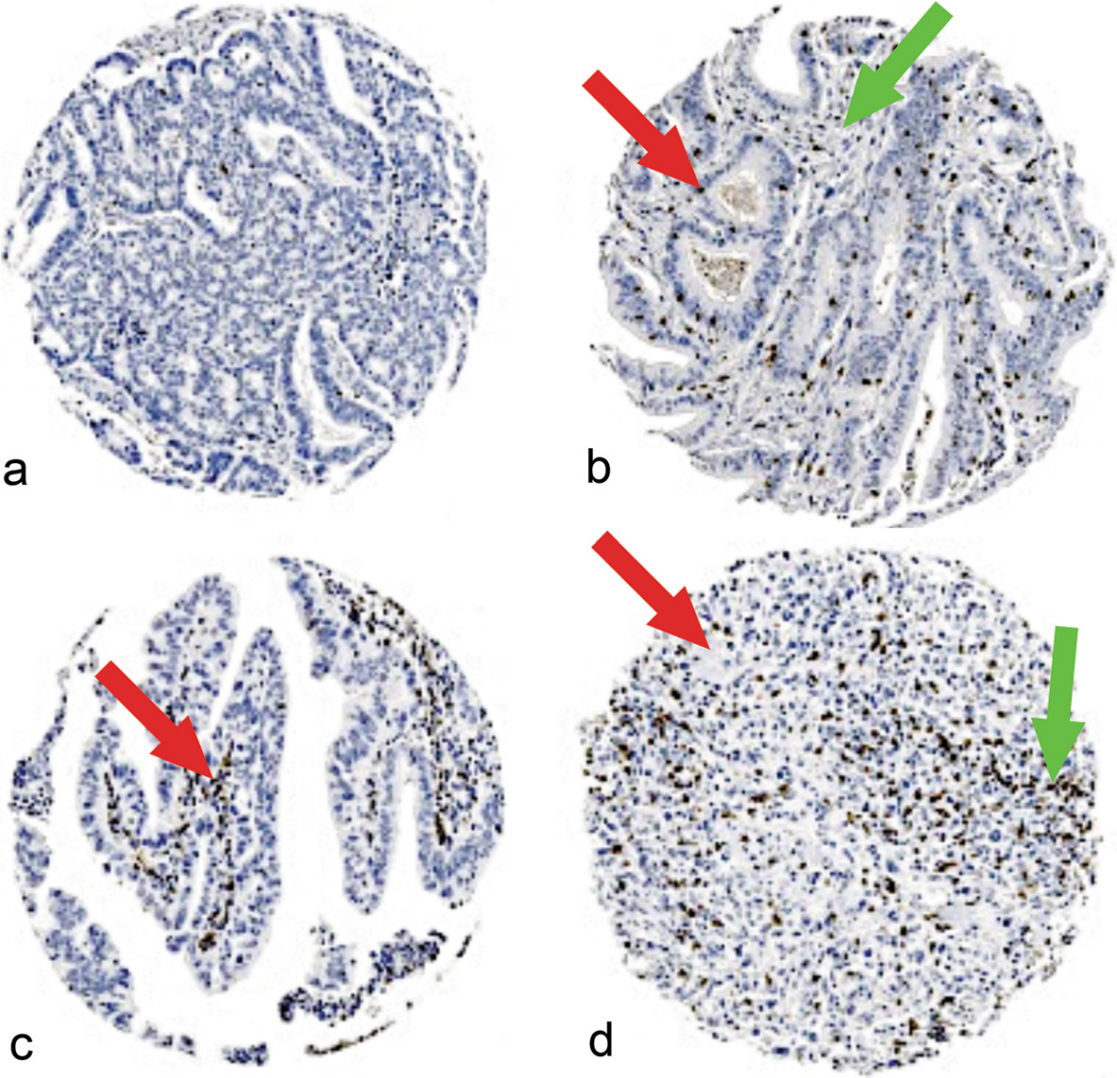
**Expression of CD8 in pancreatic ductal adenocarcinoma.** Representative photomicrographs of CD8 stained lymphocytes in pancreatic ductal adenocarcinoma. **a**: Occasional CD8 lymphocytes in tumour and stroma in moderately differentiated pancreatic adenocarcinoma (G2). **b**: Small number of CD8 lymphocytes in tumour epithelium (red arrow head) and stroma (green arrow head) of moderately differentiated pancreatic adenocarcinoma (G2). **c**: Occasional number of CD8 lymphocytes in tumour epithelium and moderate number in stroma (red arrow head) of papillary (well differentiated) pancreatic adenocarcinoma (G1). **d**: Small number of CD8 lymphocytes in tumour (red arrow head) and moderate number in stroma (green arrow head) of poorly differentiated pancreatic adenocarcinoma (G3). Photomicrographs are at 5× magnification.

**Figure 3:**
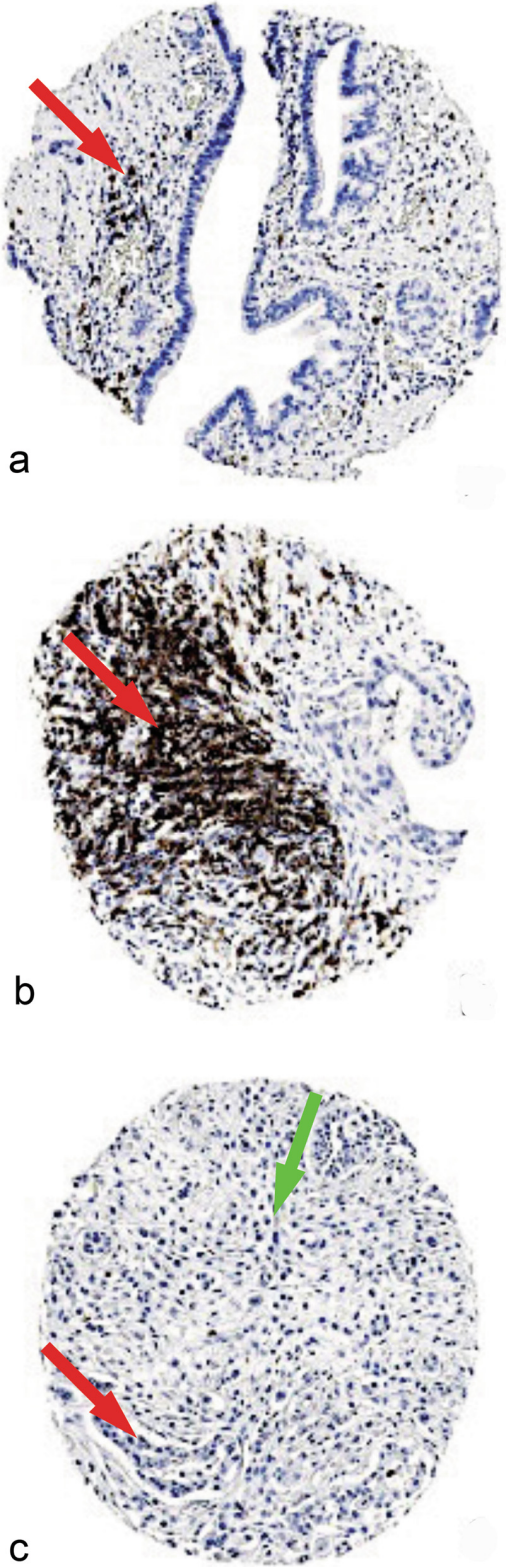
**Expression of CD20 in pancreatic ductal adenocarcinoma.** Representative photomicrographs of CD20 expression. **a**: moderate number of B lymphocytes in the stroma (red arrow head) in pancreatic carcinoma. **b**: Lymphoid follicle (red arrow head) rich in CD20 lymphocytes from patient with pancreatic carcinoma. **c**: absence of CD20 in both epithelial component (red arrow head) and stroma (green arrow head) in poorly differentiated pancreatic adenocarcinoma (G3). Photomicrographs are at 5× magnification.

CD3 staining was seen in both the tumour tissue and stroma of pancreatic adenocarcinomas (Figure 1a-d) and each was scored separately. Similarly, CD8 staining was seen in tumour and stroma (Figure 2a-d) and each was scored separately. CD20 staining was seen primarily in the stroma (Figure 3a). Staining was also seen in lymphoid follicles (Figure 3b). In poorly differentiated pancreatic adenocarcinomas, there was little positive CD20 staining in either the tumour or stroma (Figure 3c).

### Survival analysis

**Figure 4:**
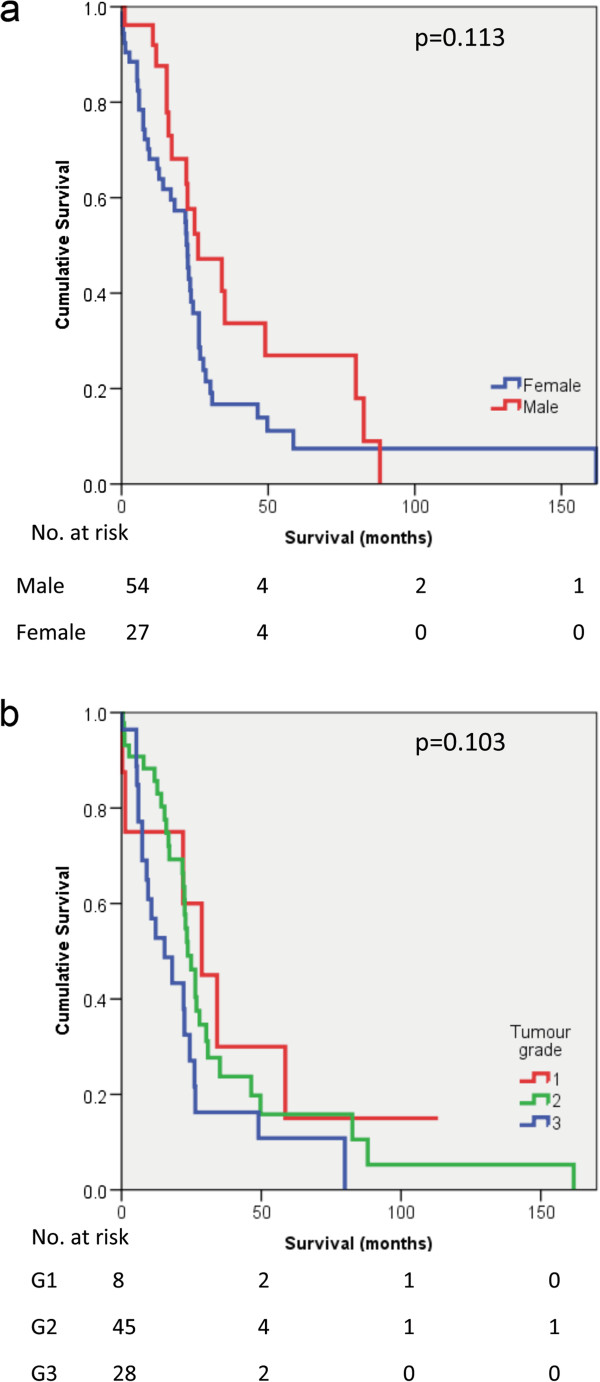
**Association of survival and patient/ tumour characteristics.** Kaplan Meier survival curves showing survival associated with **a**: gender - males (n=54) and females (n=27) and **b**: grade of tumour differentiation: G1 – well differentiated (n=8), G2 – moderately differentiated (n=45), G3 – poorly differentiated (n=28).

**Figure 5:**
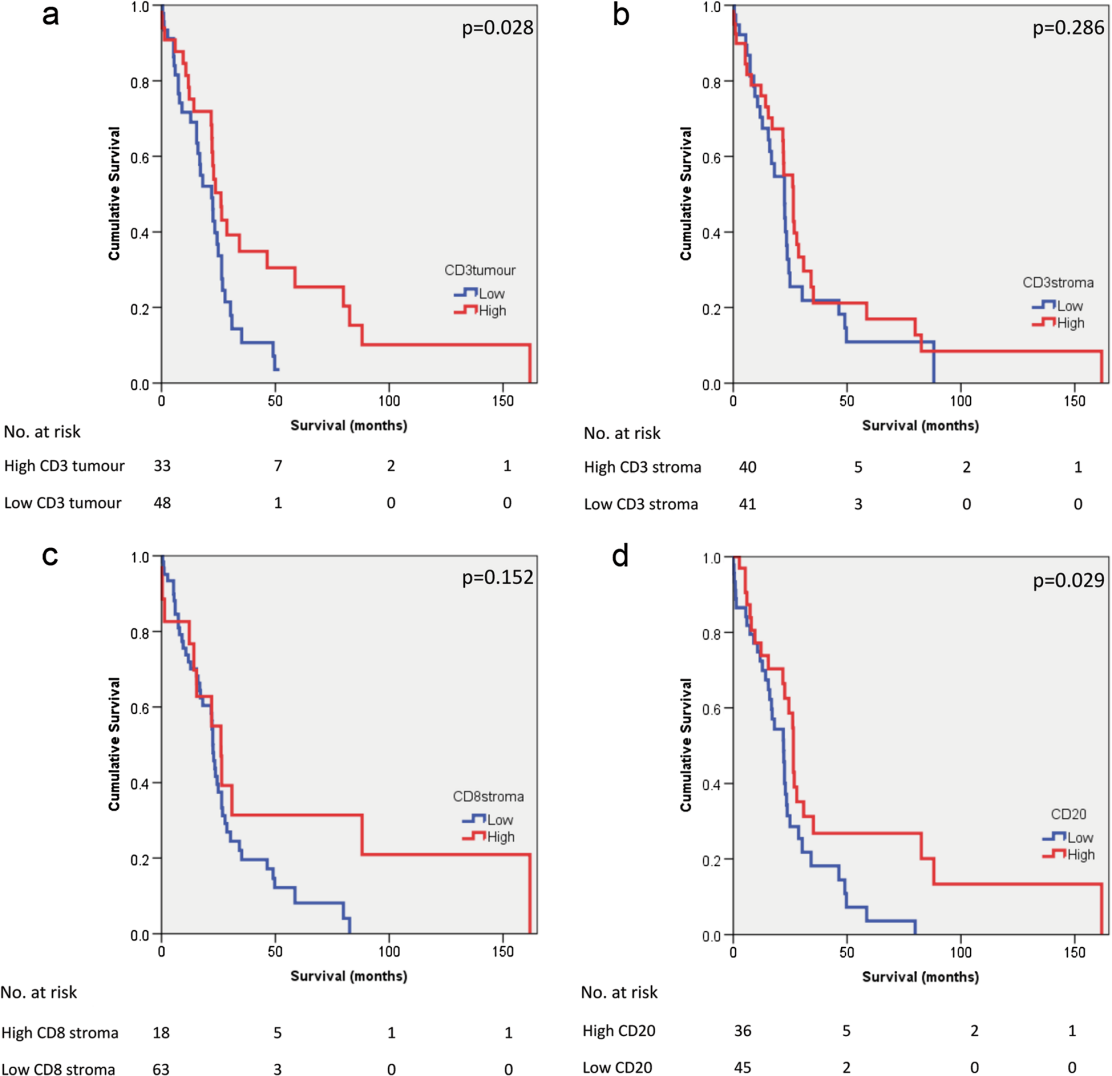
**Association of survival and tumour associated lymphocytes.** Kaplan Meier survival curves comparing patients with pancreatic ductal adenocarcinoma **a**: where intratumoural CD3 was high (N=33) *vs*. low (N=48) **b**: where stromal CD3 was high (N=40) *vs*. low (N=41) **c**: where stromal CD8 was high (N=18) *vs*. low (N=63) **d**: where CD20 was high (N=36) *vs*. low (N=45).

**Figure 6:**
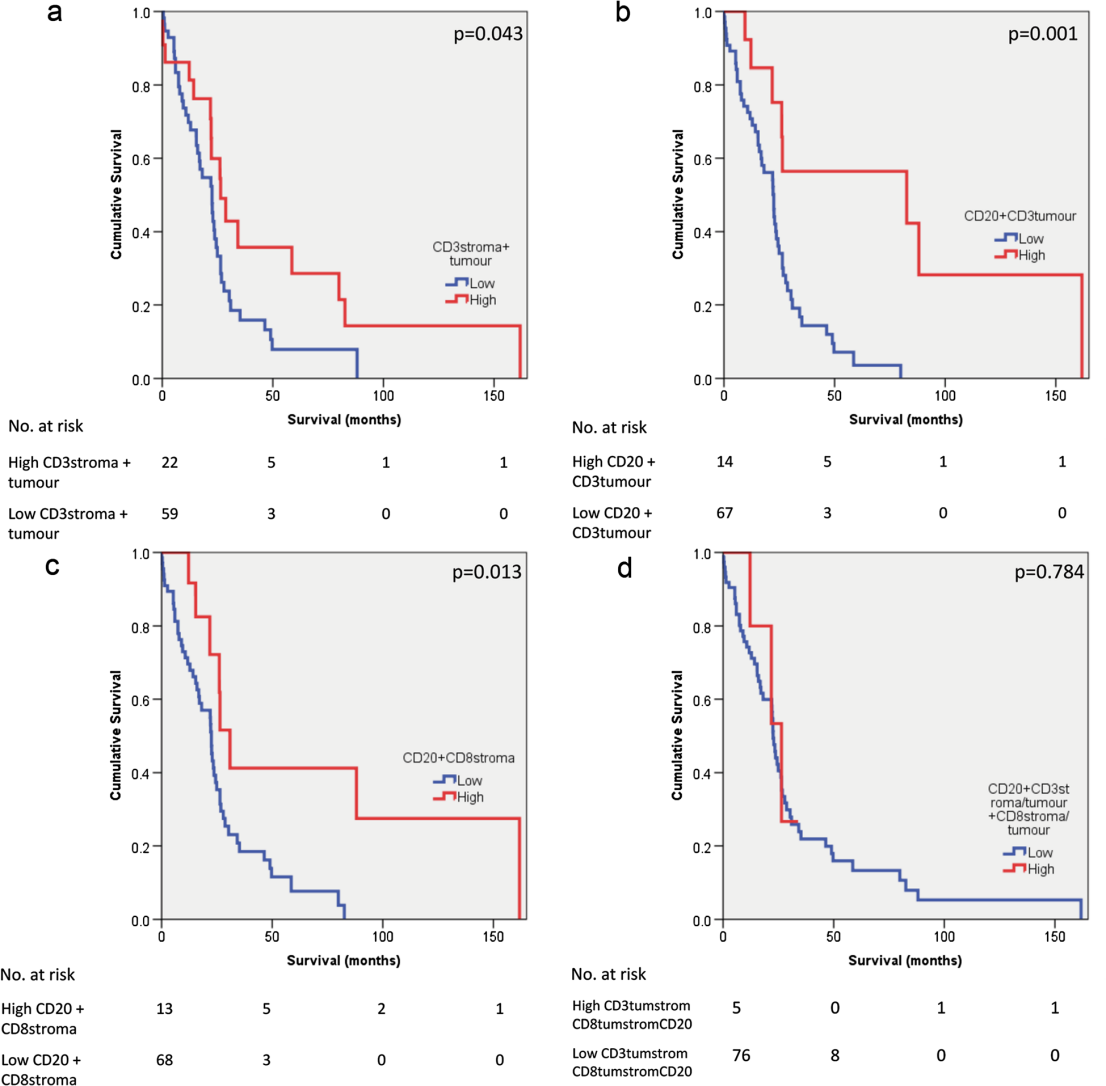
**Association of survival and tumour associated lymphocytes.** Kaplan Meier survival curves comparing patients with pancreatic ductal adenocarcinoma **a**: where intratumoural CD3 and stromal CD3 were high (N=22) *vs*. low (N=59) **b**: stromal CD3 and CD20 were high (N=14) *vs*. low (N=67) **c**: stromal CD8 and CD20 were high (N=13) *vs*. low(N=68) **d**: where stromal and intratumoural CD3, stromal and intratumoural CD8 and CD20 were all high (N=5) *vs*. low (N=76).

There was no association between sex of patients and survival (p = 0.113) (Figure 4a). There was also no significant association between tumour grade and survival (p = 0.103) (Figure 4b). Kaplan Meier plots of survival in pancreatic ductal adenocarcinoma in the presence of TALs alone, or in combination, are shown in Figure 5 (a-d) and Figure 6 (a-d). The presence of intratumoural CD3 correlated with improved survival (p = 0.028). When intratumoural CD3 and stromal CD3 were present, survival was improved (p = 0.043). The presence of CD20 positive lymphocytes correlated with improved survival (p = 0.029). When intratumoural CD3 and CD20 were present and stromal CD8 and CD20 were present, survival appeared improved (p = 0.001 and p = 0.013 respectively).

### Regression analyses

Cut-offs values for each of the clinicopathological features were determined using ROC curves. A number of prognostic factors including presence of venous invasion, perineural invasion, tumour size, grade of differentiation and lymph node status (positive or negative for tumour) were tested to determine whether CD3, CD8 and CD20 positive lymphocytes in the tumour or stroma were related to them.

In pancreatic ductal adenocarcinoma, the presence of intratumoural CD3 was significantly associated with grade of tumour differentiation (p = 0.049). Stromal CD8 also correlated with grade of tumour differentiation (p = 0.015). The presence of stromal CD3 and CD8 in combination, and stromal and intratumoural CD3 in combination also correlated with grade of tumour differentiation (p = 0.049 and p = 0.010 respectively). The only other positive correlation was between the presence of intratumoural CD8 and CD20 in combination with perineural invasion (p = 0.048). Table 3 shows all the correlations tested with their results.

**Table 3:** Regression results (p values) of all variables tested and TALs

	**Lymph node status**	**Grade of differentiation**	**Tumour size**	**Venous invasion**	**Perineural invasion**	**Survival**
CD3 tum	0.545	0.049	0.155	0.631	0.228	0.028
CD3 strom	0.165	0.054	0.749	0.753	0.746	0.286
CD8 tum	0.755	0.876	0.224	0.734	0.290	0.289
CD8 strom	0.329	0.015	0.081	0.257	0.889	0.152
CD3tumstrom	0.792	0.010	0.206	0.724	0.278	0.043
CD8tumstrom	0.608	0.937	0.226	0.598	0.441	0.967
CD3tum + CD8tum	0.832	0.567	0.380	0.711	0.777	0.624
CD3strom + CD8strom	0.109	0.049	0.300	0.544	0.745	0.377
CD3tumstrom + CD8tumstrom	0.836	0.863	0.374	0.368	0.159	0.712
CD20	0.514	0.057	0.736	0.635	0.446	0.029
CD20 + CD3tum	0.066	0.758	0.443	0.299	0.844	0.001
CD20 + CD3 strom	0.458	0.441	0.883	0.234	0.290	0.144
CD20 + CD8 tum	0.060	0.288	0.231	0.646	0.048	0.706
CD20 + CD8 strom	0.154	0.640	0.245	0.134	0.143	0.013
CD20 + CD3tumstrom + CD8tumstrom	0.125	0.582	0.421	0.514	0.201	0.784

tum = intratumoural, *strom* stromal, tumstrom = intratumoural and stromal.

## Discussion

In this study we have evaluated the prognostic significance of TALs in pancreatic ductal adenocarcinomas. This cancer generally presents at an advanced stage and is associated with poor prognosis. In cancer patients, the identification of markers which could predict survival or risk of metastases would be useful [22]. The finding of a correlation between the presence of CD3 positive lymphocytes in the tumour tissue and improved survival in pancreatic ductal adenocarcinoma is promising. In addition, the combination of tumoural CD3 and CD20 as well as stromal CD8 and CD20 correlated with survival. This bodes well for the development of potential therapeutic targets, although it may take a significant length of time. Although there are a number of indicators of outcome available, such as nodal involvement and resection margin, the addition of further markers such as the presence of tumour associated lymphocytes may allow us to provide additional useful information to the patient regarding their prognosis.

This study demonstrated that the presence of CD3 and CD8 positive lymphocytes was associated with increasing grade of tumour differentiation in pancreatic ductal adenocarcinoma. Although we did not demonstrate a significant association between tumour grade and survival, it is well established that poorly differentiated pancreatic cancers are associated with poorer survival [23]. Our results suggest that well differentiated tumours may be associated with a more aggressive immune reaction and, therefore, confer a more favourable prognosis.

There was also an association between the presence of high levels of CD20 in combination with intratumoural CD8 and perineural invasion. Perineural invasion in pancreatic cancer is associated with poor prognosis [24,25] and may be present even in the absence of lymph node metastases [26]. It is difficult to explain the finding that the presence of CD8 and CD20 positive lymphocytes correlated with other known poor prognostic markers, such as perineural invasion but also correlated with improved survival. In other solid tumours, such as thyroid cancer, the presence of TALs has been associated with more aggressive disease [27]. However, in colorectal cancer, the presence of TALs signifies an inflammatory cell reaction at the tumour invasive border and appears to be a useful predictor of survival [28].

This study also evaluated CD20, a marker for B lymphocytes, in pancreatic cancer. CD20 positive B lymphocytes have been recently evaluated in advanced gastric cancer [29] and were found not to be associated with prognosis in prostate cancer [30]. The novel finding of a positive association between CD20 positive B lymphocytes and survival in pancreatic ductal adenocarcinoma merits further investigation.

## Conclusions

In summary, our study evaluated the presence of CD3, CD8 and CD20 positive lymphocytes in a large series of surgically resected pancreatic ductal adenocarcinoma and demonstrated correlation with survival in pancreatic cancer, which may be related to lymph node metastases, perineural invasion or tumour size. One of the limitations of this study is the use of TMAs which may not be representative of the whole tumour specimen because of tissue heterogeneity. However, the use of TMA cores constructed in triplicate has been shown to provide a sufficient level of sampling uniformity [31,32]. Although archival specimens were used, previous studies have suggested that many proteins are antigenically retrievable on tissues stored for more than 60 years [33]. In conclusion, this study of TALs has shown that they are associated with improved survival in pancreatic ductal adenocarcinoma. The surprising finding of a positive association between CD20, intratumoural CD8 and perineural invasion was also reported. Whether this has a bearing on survival is not clear from our results. Future studies are needed to confirm these results in an independent data set and to elucidate the exact mechanisms of a lymphocytic reaction to tumour.

## Competing interests

The authors declare that they have no competing interest.

## Authors’ contributions

Design of study: NT, AMZ, AA, SM, MI, DNL. Data collection: NT, AMZ, AA. Analysis and interpretation of results: NT, AMZ, AA, SM, MI, DNL. Drafting and editing of manuscript: NT, AMZ, AA, SM, MI, DNL. Final approval: NT, AMZ, AA, SM, MI, DNL. All authors read and approved the final manuscript.

## Pre-publication history

The pre-publication history for this paper can be accessed here:

http://www.biomedcentral.com/1471-2407/13/436/prepub
